# Quantitative probe for in-plane piezoelectric coupling in 2D materials

**DOI:** 10.1038/s41598-021-86252-9

**Published:** 2021-03-29

**Authors:** Sai Saraswathi Yarajena, Rabindra Biswas, Varun Raghunathan, Akshay K. Naik

**Affiliations:** 1 Centre for Nano Science and Engineering, Indian Institute of Science, Bengaluru, 560012 India; 2 Department of Electrical Communication Engineering, Indian Institute of Science, Bengaluru, 560012 India

**Keywords:** Nanoscience and technology, Nanoscale materials, Techniques and instrumentation

## Abstract

Piezoelectric response in two-dimensional (2D) materials has evoked immense interest in using them for various applications involving electromechanical coupling. In most of the 2D materials, piezoelectricity is coupled along the in-plane direction. Here, we propose a technique to probe the in-plane piezoelectric coupling strength in layered nanomaterials quantitively. The method involves a novel approach for in-plane field excitation in lateral Piezoresponse force microscopy (PFM) for 2D materials. Operating near contact resonance has enabled the measurement of the piezoelectric coupling coefficients in the sub pm/V range. Detailed methodology for the signal calibration and the background subtraction when PFM is operated near the contact resonance of the cantilever is also provided. The technique is verified by estimating the in-plane piezoelectric coupling coefficients (*d*_11_) for freely suspended MoS_2_ of one to five atomic layers. For 2D-MoS_2_ with the odd number of atomic layers, which are non-centrosymmetric, finite *d*_11_ is measured. The measurements also indicate that the coupling strength decreases with an increase in the number of layers. The techniques presented would be an effective tool to study the in-plane piezoelectricity quantitatively in various materials along with emerging 2D-materials.

## Introduction

2D materials including transition metal dichalcogenides (TMDCs), hBN exhibit in-plane piezoelectricity^1–3^. Recent reports on 2D Janus TMDCs also indicate large out of the plane and in-plane piezoelectricity^4^. This has relevance in many applications such as sensing^5^, energy harvesting^6,7^, and piezotronics^8^. The coefficient *d*_11_ is the standard parameter to estimate the strength of the in-plane piezoelectric coupling^9^. The subscript ‘11’ in the notation refers to the piezoelectric-coupling tensor element where the applied electric field and the displacement are along the crystallographic x-direction of the material. For example, *d*_11_ alone can describe the complete piezoelectric tensor for a monolayer MoS_2_ (Molybdenum disulfide). The non-zero elements in MoS_2_ piezoelectric-coupling tensor are *d*_11_, *d*_12_ and *d*_*26*_ where *d*_11_ =  − *d*_12_ =  − *d*_26_/2*.* Here, crystallographic ‘x’ refers to the armchair direction^1,10^.

Zhu et al.^11^ have experimentally verified the piezoelectric response in an odd number of layers of MoS_2_. They have calculated piezoelectric stress coefficients (*e*_11_) using the nano-indentation method and have indicated that lateral PFM cannot be performed on MoS_2_. The method used to estimate *e*_11_ requires a dedicated setup and specialized fabrication process to modify the commercial AFM (Atomic Force Microscopy) tips. Esfahani et al*.*^12^ have attempted lateral field excitation for 2D materials. Still, the study is limited to measuring out-of-the-plane displacement using vertical PFM. That phenomenon is called as flexoelectricity. Various studies conducted by Wu et al*.*^13^, Qi et al*.*^14^, Zelisko et al*.*^15^, and Wang et al*.*^16^ have verified piezoelectric behaviour in some of the 2D materials. However, these studies on the in-plane piezoelectric coupling are limited to the qualitative discussion. Thus, a robust and simple technique is needed to measure in-plane piezo coupling coefficients for 2D- materials.

We propose the complete scheme of measurement to estimate the in-plane piezoelectric coupling coefficients (*d*_11_). The methods allow us to perform lateral PFM on 2D materials with in-plane excitation. These measurements can be performed on most commercially available AFMs using commercial AFM tips. PFM is one of the application modules in AFM, which is utilized to characterize the piezoelectric and ferroelectric properties of the material^17^. It is widely used for the measurement of piezo coupling coefficients^18–20^. The piezoelectric coupling coefficient is calculated as displacement per unit applied voltage referred to as ‘*d*’ coefficients^21^. Most commercial AFMs are equipped with four-quadrant position-sensitive photodetectors, enabling the measurement of both lateral and vertical displacement of the tip. In the vertical mode of PFM, the cantilever's vertical displacement causes a vertical deflection signal, whereas in the lateral mode, the torsional bending of the cantilever causes a lateral deflection signal. In lateral PFM, calibrated torsion of the cantilever caused by in-plane piezoelectric effect is used to measure the effective lateral displacement^22,23^. Furthermore, lateral PFM measurements can be performed with electric field excitation in out-of-plane as well as in-plane. Combinations of possible electric field excitation and detection displacement directions allow us to probe different coupling elements in piezoelectric tensor corresponding to the material's crystal structure. Hence to measure the *d*_11_ coefficient for 2D materials, lateral PFM with in-plane field excitation is required.

2D materials have relatively low coupling coefficients^1,4^. In the current study, contact resonance gain of the AFM cantilever is leveraged to improve the detection sensitivity by more than an order of magnitude, which enabled the measurement of the piezoelectric coefficients in the range of a few pm/V to sub pm/V feasible. In any AFM related measurements, the detection scheme involves the extraction of the information from tip-sample interactions. As an electrically conductive AFM tip is used for the measurements, various electrostatic interactions affect the measured response^24,25^. Here, we demonstrate simple ways to quantify and eliminate the contribution from background signals involved in the current measurement scheme. We also present discussion on the selection of AFM tips and the effect of local electrostatic forces related to the stiffness of the cantilever. Measurements have been carried out for MoS_2_ flakes of thickness ranging from one atomic layer to 5 atomic layers. It is verified that even number of layers do not exhibit piezoelectricity because they lack centrosymmetry.

## Results and discussions

**Figure 1:**
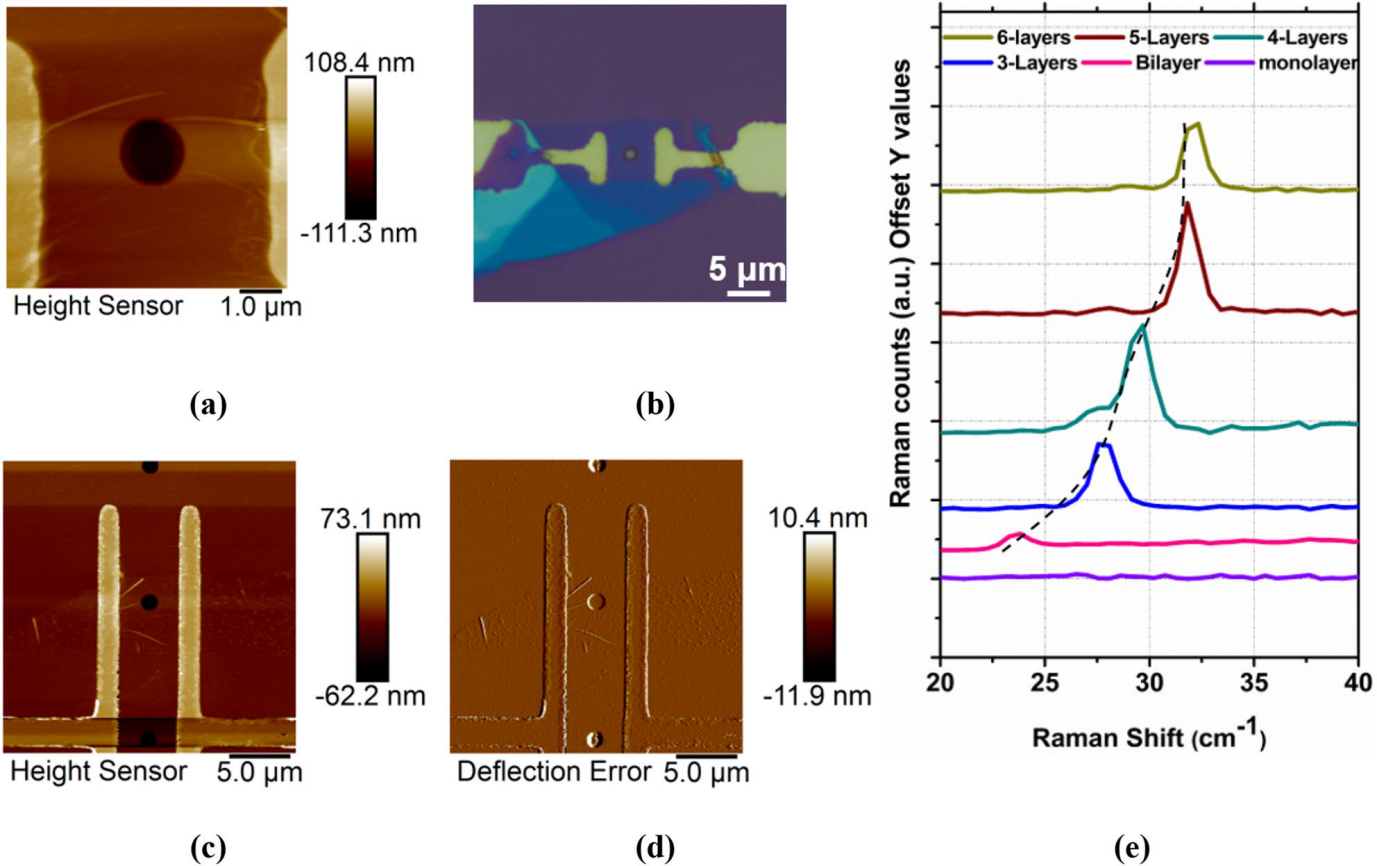
(**a**) AFM topography image of the suspended monolayer MoS_2_ device-1. (**b**) Optical micrograph of device-1. (**c**), (**d**) AFM topography and deflection error image of device-2 (monolayer MoS_2_) respectively showing that the flake is suspended on one of the circular trenches. (**e**) Low-frequency Raman modes of MoS_2_ flakes with 1–6 layers.
B. **Proposed scheme of measurement**
  Figure 2a shows the schematic of the measurement system to perform lateral PFM with in-plane field excitation. An internal lock-in amplifier (with an amplification factor *g*_*Lin*_) is employed for PFM measurements in the AFM systems to measure the lateral deflection. From the position-sensitive photodetector (PSPD), lateral deflection information [(A + C) − (B + D)] can be acquired. The PFM system generates an amplitude signal A(ω) in response to the lateral deflection of the cantilever. Lateral deflection corresponding to the amplitude can be measured using the lateral deflection sensitivity factor (*l*_*d*_). We have opted for the angle conversion factor method proposed by Choi et al.^31^ to calculate the lateral deflection sensitivity. Here the lateral twist angles of the cantilever when an AFM tip is climbing up the surface as depicted in Fig. 2b are correlated with the PSPD's voltage readings for the calibration of *l*_*d*_ (detailed procedure can be found in Sects. 4 and 5 of SI).

A. **Device architecture and characterization**
  Suspended MoS_2_ devices are fabricated on Si/SiO_2_ substrates (detailed fabrication is included in Sects. 1 and 2 of SI) for the PFM measurements. Substrate effects such as charge screening and doping can influence the piezoelectric response^11,16^. To avoid these effects, MoS_2_ is suspended at the point of measurement. Figure 1a–d shows the AFM and optical topography image of the suspended monolayer MoS_2_ devices. Thin flakes are identified based on optical contrast using an optical microscope. The number of layers in MoS_2_ flakes are confirmed using Raman spectroscopy based on the peak positions of low-frequency shear vibrational mode (S_1_) and in-plane vibrational modes (E_2g_ and A_1g_)^26^. The position of low-frequency shear vibrational modes can be utilized to identify up to 6 layers of MoS_2_^27^ (Fig. 1e). These flakes are transferred onto the substrate, using dry transfer method^28^, onto a pre-patterned Si/SiO_2_ substrate with Ti/Pt (Ti-Titanium, Pt-Platinum) metal electrodes and a circular trench in between them. The electrodes are referred to as the source and drain contact electrodes in further discussion. The edge chirality of these exfoliated flakes is determined by second harmonic generation microscopy (SHG)^29,30^. Angle dependent SHG study (see Sect. 3 of SI) shows that the electrodes are placed in the armchair direction of the monolayer MoS_2_ flakes.

**Figure 2:**
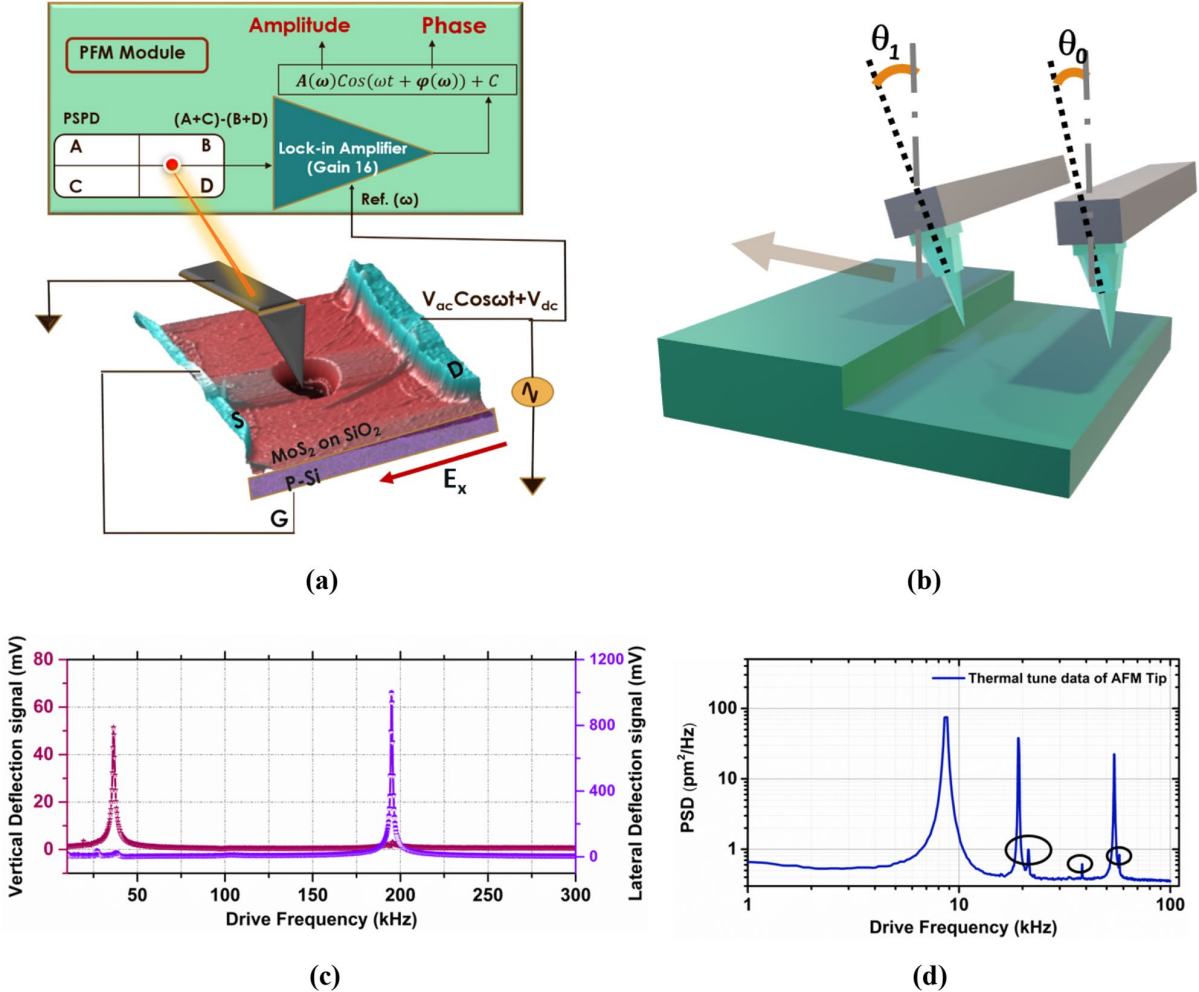
(**a**) Schematic of lateral PFM system with in-plane excitation (S, D, and G refer to the source, drain and gate respectively, p-Si refers to a silicon substrate with acceptor doping/p-type and dimensions not to the scale). (**b**) Schematic explaining angle conversion factor method for lateral deflection sensitivity calibration (arrow indicates the direction of scanning). (**c**) The vertical and lateral frequency response of the AFM cantilever when the tip is in contact with the sample. (**d**) Power spectral density (PSD) during the thermal tuning of AFM tip (free vibrations). The circled regions indicate the electronic noise peaks related to the system.
  The AFM tip is placed on the suspended portion of the MoS_2_ at a constant normal force to carry out measurements. For the in-plane field excitation, the voltage signals are applied to the drain electrode (Fig. 2a). Potential difference is applied between the electrode and the AFM tip, which is in contact with the sample. As the AFM tip is at ground potential, the direction of the electric field in the sample is from the drain electrode to the point of contact of the tip. This direction is the crystallographic x-direction of MoS_2_ as determined using the SHG measurement (see SI). The source and gate electrodes are connected to the external ground to avoid the coupling in other directions.
  There are alternate ways to apply the in-plane electric field, viz.; (A) applying voltage signal between the source and the drain electrodes while the tip is placed on suspended MoS_2_ drum region, and (B) applying the voltage signals to the tip and keeping the drain at ground potential. However, both of these configurations are suboptimal. In configuration A, the lateral displacement of the AFM tip due to the in-plane piezoelectric effect is zero because MoS_2_ stretches uniformly in both directions when the tip is placed in the centre (refer Sect. 6 of SI). In configuration B, there would be a potential difference between the AFM tip placed on suspended MoS_2_ and the gate electrode (doped silicon underneath the MoS_2_) in the vertical direction. The measured piezoelectric response would have a vertical response coupled with the lateral piezoelectric response. Hence, to detect the finite lateral deflection and the measurement accuracy, we have opted for the configuration as shown in Fig. 2a.
  The choice of the AFM tip for the measurements is critical as the measurements are carried out on the freely suspended layers of sub-nm to few nanometres in thickness. When the tip is abruptly placed on the suspended region with high normal forces, the suspended layer/s suffer from peak stress and thus collapse. Hence, a compliant cantilever with a normal spring constant of 0.1–0.2 N/m is used in these measurements (SCM-PIC V2 tip; refer to methods). The applied normal force is maintained between 30–40 nN. In PFM measurements of samples with vertical domains, cantilevers with small spring constants are a disadvantage because of the large electrostatic contribution^24,32,33^. However, since all our PFM measurements are in lateral direction, the small spring constant in normal direction is not an impediment. Furthermore, the lateral spring constant of cantilevers used in our experiments are in the range of 25–35 N/m. This large lateral spring constant, as explained below, ensures that there are minimal electrostatic contributions to our lateral PFM measurements.
  At resonance, the motion of the cantilever is amplified by the quality factor and the displacement sensitivity is improved (refer Sect. 7 of SI for instrument background noise). The frequency response is obtained when the AFM tip is placed in contact with the suspended MoS_2_ region. Figure 2c shows these frequency responses of lateral and vertical displacements of the AFM cantilever. These are obtained by monitoring the signals from 4 quadrant PSPD. From this, lateral and vertical contact resonance frequency ranges are identified for the tip-sample system. During the measurement, the applied normal force is kept constant. Typical lateral piezoelectric coupling coefficients for 2D materials are in the range of sub pm/V to few pm/V. By choosing the operation frequency of lateral PFM near to the torsional contact resonance (referred to as contact resonance in further discussion on lateral PFM), we can leverage the gain provided by the quality factor and make picometre scale lateral deflection measurements feasible.
  The frequency range of vertical resonance is initially identified from the free cantilever vibrations of the cantilever called the thermal tuning data (Fig. 2d). The normal resonance frequency of the SCM-PIC tip (refer methods) is around 10 kHz, and the other peaks are the higher-order harmonics. Drive frequency of operation is chosen such that the electronic noise of the system does not fall in this frequency range. These noise peaks (circled peaks in Fig. 2d) and their corresponding frequencies are identified from the thermal tuning data of the free cantilever. While performing lateral PFM measurements, the background noise can be reduced by avoiding the frequencies near vertical resonance and other noise peaks. Since there is a large difference between vertical and lateral spring constants, the contact resonance frequency peaks for the vertical and lateral deflections are far apart. This reduces the cross-coupling effects between lateral and vertical deflection signals.
  The voltage signal detected by the lock-in amplifier from the lateral deflection signal is $A_{l} \left( \omega \right)$ for the applied drive signal *V*_*ac*_* cos(ωt),* where *ω* is the drive frequency, and *V*_*ac*_ is the drive amplitude. The piezoelectric response $PR_{l} \left( \omega \right)$ corresponding to lateral deflection is measured from the lateral deflection sensitivity factor ($l_{d}$) is $l_{d} A_{l} \left( \omega \right)$ (Response is corrected for the factor of lock-in gain *g*_*Lin*_). Figure 3a shows the lateral piezoelectric response measured on the suspended MoS_2_ sample near the contact resonance frequency. The slope of the piezoresponse $PR_{l} \left( \omega \right)$ is called the measured *d*_11_ coefficient (*d*_11*meas*_).
  1 $$PR_{l} \left( \omega \right) = d_{11meas} V_{ac}$$

**Figure 3:**
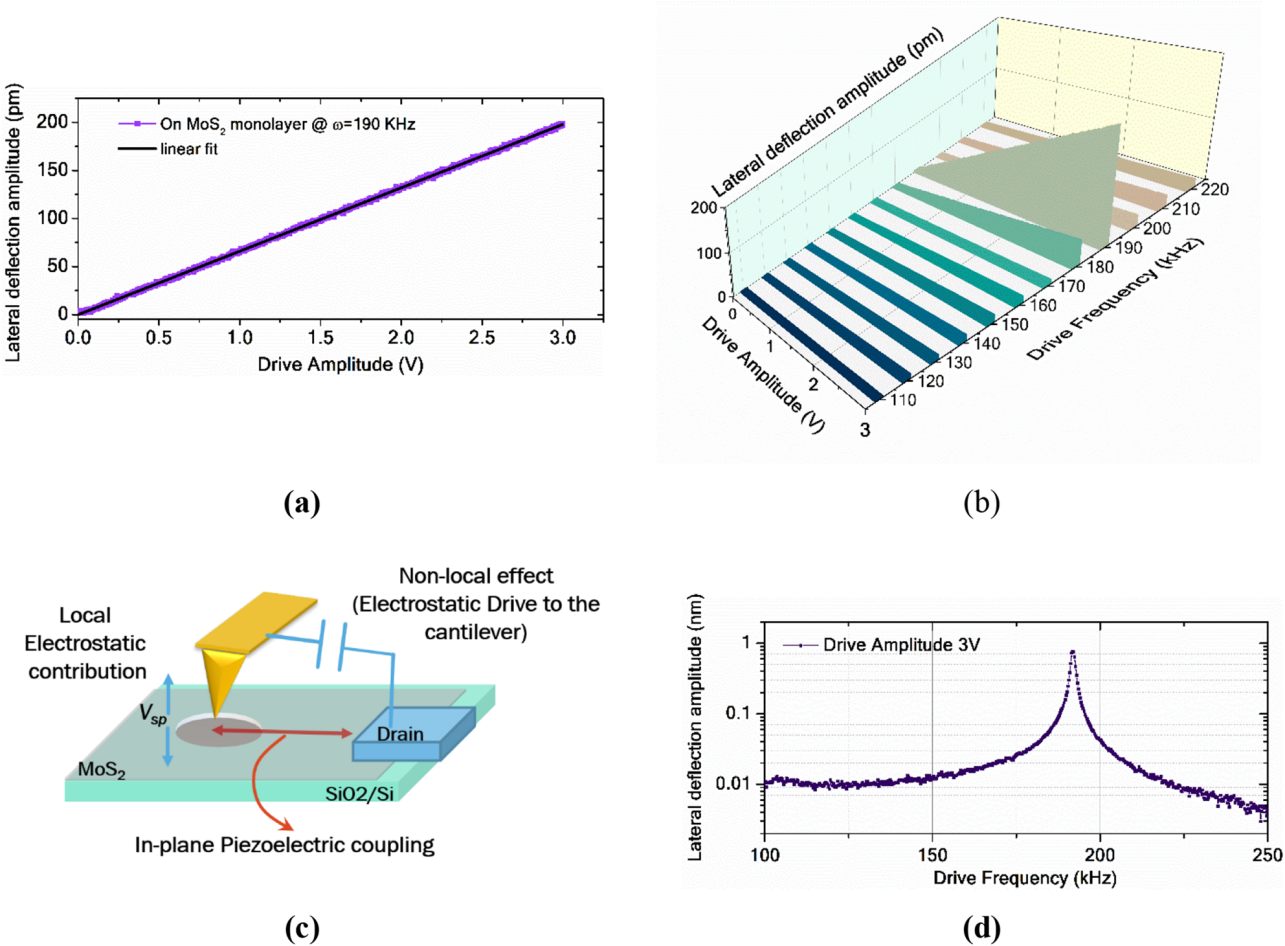
(**a**) Piezoelectric response measured close to contact resonance of the cantilever. (**b**) Lateral piezo response as the frequency of measurement is varied around the contact resonance frequency. (**c**) Schematic showing various contributions to the signal measured (Dimensions are not to the scale). (**d**) Lateral piezoelectric response on 1L-MoS_2_ observed at variable drive frequencies at constant ac drive.
  Figure 3b shows the piezoelectric response obtained at different frequencies near contact resonance, from which it can be observed that the measured response is much larger near the resonance frequency. Also, there is a significant amount of background signal in the current measurement scheme due to local electrostatic effects and contact resonance of the cantilever. At a given frequency of operation, we have identified three major contributions to the measured lateral piezoelectric response viz. (a) actual in-plane piezoelectric response of the material $\left( {PR_{piezo} } \right)$, (b) the net non-local electrostatic contribution from the cantilever resonance, which we term as pseudo piezoelectric response $\left( {PR_{pseudo} } \right)$ and (c) the local electrostatic response $(EL_{l} )$ which has its origin in the finite surface potential^24^. These contributions are represented in Fig. 3c (just for illustration). The measured lateral piezoelectric response [Eq. (1)] is thus related to these contributions as follows
  2 $$PR_{l} \left( \omega \right) = PR_{piezo} \left( \omega \right) + PR_{pseudo} \left( \omega \right) + EL_{l} \left( \omega \right)$$
C. **Extraction of effective** ***d***_***11***_ **coupling coefficient**
  To extract the effective piezoelectric coefficient (*d*_11*eff*_) from the measured response [Eq. (2)], we need to estimate all background signals quantitatively. The gain in the signal by operating near the resonance $g_{PR} \left( \omega \right)$ can be calculated using the frequency response curves (Fig. 3d) and is formulated in Eq. (3),
  3 $$g_{PR} \left( \omega \right) = [PR_{l} \left( \omega \right)/PR_{l} \left( {\omega_{b} } \right)]\,At\,constant\,V_{ac}$$
  where *ω*_*b*_ is the frequency far from the resonance frequency (100 kHz in this case and referred to as base frequency, refer to Sect. 8 of SI for more details).
  The drive signal (*V*_*ac*_* cos*(*ωt*)) is the electrostatic drive to the cantilever. Increasing the drive amplitude (*V*_*ac*_) enhances the contact resonance gain at a given drive frequency. Figure 4a shows the frequency (*ω*) response curves near contact resonance at variable drive amplitudes (*V*_ac_). The increase in the piezoelectric response with *V*_*ac*_ is the combined effect of the piezoelectric effect in the sample and the electrostatic drive on the cantilever system. The electrostatic drive causes a finite lateral response on the non-piezoelectric material when operated at the contact resonance frequency. This effect is a non-local electrostatic contribution as the effect is not from the point of measurement on the sample.

**Figure 4:**
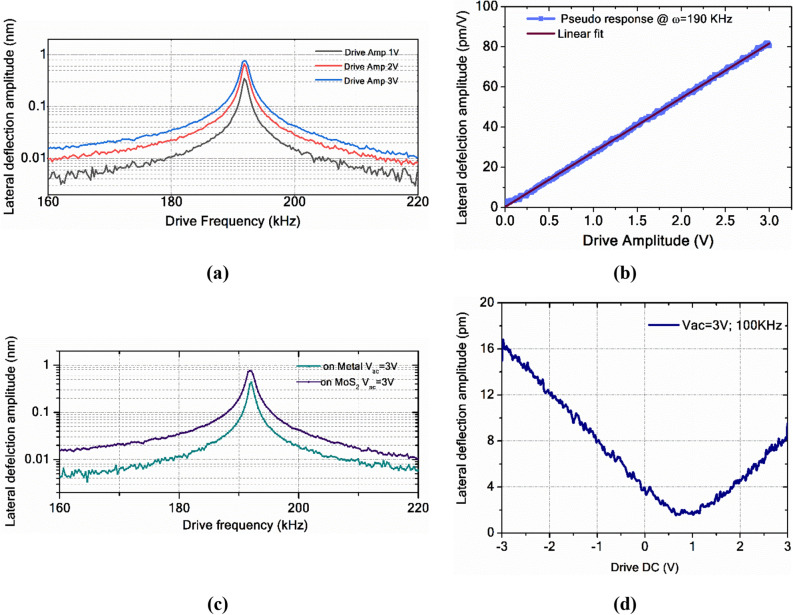
(**a**) Frequency response curves of lateral piezoresponse at different drive amplitudes showing the tuning of contact resonance with applied drive amplitude (*V*_*ac*_). (**b**) Pseudo-piezoelectric response on the metal electrode near contact resonance. (**c**) Lateral frequency response curves on the metal (source electrode) and suspended MoS_2_ near contact resonance at Vac = 3 V. (**d**) Lateral piezoelectric response on MoS_2_ at different DC voltages.
  To quantify the contribution from electrostatic drive near contact resonance frequency, we propose a method involving a separate set of measurements called pseudo-piezoresponse measurements. Pseudo piezoresponse refers to the cause of the lateral displacement of the conductive AFM tip upon in-plane excitation on one of the electrodes. We call it pseudo-piezoresponse as it gives the piezoelectric kind of response on non-piezoelectric samples when PFM is operated at the resonance. Figure 4b shows the pseudo piezoresponse measured on the metal (source electrode), which is not a piezoelectric material. These measurements can be performed on the same sample. Lateral deflection response is obtained on the source electrode, where the in-plane field excitation is from the source to the drain electrodes under the same operating conditions. Figure 4c compares the frequency response curves obtained on the source electrode (pseudo-piezoresponse) and the suspended monolayer MoS_2_ near contact resonance. Measured pseudo piezoelectric response on a metal (source electrode) is termed as
  4 $$PR_{pseudo} \left( \omega \right) = g_{pseudo} \left( \omega \right)\left[ {d_{pseudo} .V_{ac} } \right]$$
  where $d_{pseudo}$ is the pseudo coupling coefficient, it provides an estimate of the change in frequency response with applied drive signal (*V*_*ac*_) in the absence of the piezoelectric effect. The resonance gain $g_{pseudo} \left( \omega \right)$ for the pseudo-response is calculated independently on the metal (source electrode) as $[PR_{pseudo} \left( \omega \right)/PR_{pseudo} \left( {\omega_{b} } \right)]$, where *ω*_*b*_ is the base frequency of the drive frequency response on the metal electrode. It is to be noted that the normal force applied on the tip must be maintained constant for all the piezo and pseudo piezo measurements to ensure that the elastic strength is the same when the tip is in contact with the material across all the measurements. In the current set of measurements, estimated $d_{pseudo}$ near the contact resonance (190 kHz), is measured to be about 0.8 pm/V (Sect. 9 of SI). This is a significant contribution when the *d*_*11*_ coefficients to be measured are in the range of a few pm/V.
  The finite surface potential of the material leads to local electrostatic contribution to the signal. The vertical electrostatic force because of surface potential is given by^34^ $C_{z}^{{\prime }} \cdot V_{ac} cos\left( {\omega t} \right) \cdot \left( {V_{dc} - \left| {V_{sp} } \right|} \right)$, where $C_{z}^{{\prime }}$*, V*_*ac*_*, V*_*dc*_*, V*_*sp*_ are capacitance derivative along z-axis (vertically along tip), ac drive, dc drive offset and surface potential of sample with respect to the tip, respectively. Hence in case of vertical PFM, amplitude of the local electrostatic contribution is expressed as^32^ $k_{N}^{ - 1} C_{z}^{{\prime }} \cdot V_{ac} \cdot \left| {V_{dc} - V_{sp} } \right|$, where *k*_*N*_ is the normal spring constant.
  Similarly, in the lateral PFM, the local electrostatic contribution to the lateral displacement is caused by the lateral electrostatic force. The contribution from this to the lateral piezoresponse is related to the lateral spring constant of the cantilever, and it can be expressed using the following expression.
  5 $$x_{{EL_{l} }} = k_{l}^{ - 1} C_{l}^{{\prime }} \cdot V_{ac} \cdot \left| {V_{dc} - V_{sp} } \right|$$
  where *k*_*l*_*,* $C_{l}^{{\prime }}$*, V*_*ac*_, *V*_*dc*_, and *V*_*sp*_ are lateral spring constant, capacitance derivative along the in-plane axis (lateral direction between the point of measurement and the driving electrode), ac drive voltage, dc drive offset, and surface potential of the sample with respect to the tip respectively.
  The local electrostatic contribution to the obtained piezoelectric electric response can be estimated by making V_dc_ = 0 V in Eq. (5) and it is given by $k_{l}^{ - 1} C_{l}^{{\prime }} \cdot V_{ac} \cdot \left| {V_{sp} } \right|$. To calculate the unknown term $k_{l}^{ - 1} C_{l}^{{\prime }} ,$ the lateral piezoelectric response is obtained by varying the DC offset (*V*_*dc*_). Figure 4d shows the piezoelectric response obtained with a variable DC bias field at a constant ac drive amplitude (*V*_*ac*_) and drive frequency ($\omega_{b} )$. The slope of the piezoelectric response versus drive DC offset gives the value of $k_{l}^{ - 1} C_{l}^{^{\prime}} V_{ac}$ for the given $V_{ac}$. We have observed that the contribution from the local electrostatic component to the lateral deflection is minimal (in the range of 0.05–0.1 pm/V) in the current measurements. The reasons for the minimal contribution are the large lateral spring constant of the AFM tip used (20–25 N/m) and the smaller surface potential difference for MoS_2_ (0.1–0.4 V)^35,36^.
  By extracting various contributions from Eqs. (4–9), effective in-plane piezoelectric coupling coefficient *d*_*11eff*_ can be obtained from Eq. (6).
  6 $$d_{11eff} = \frac{1}{{g_{PR} \left( \omega \right)}}\left( {\frac{{d\left( {PR_{l} \left( \omega \right)} \right)}}{{d\left( {V_{ac} } \right)}}} \right) - \frac{1}{{g_{PR\;pseudo} \left( \omega \right)}} \left( {\frac{{d(PR_{pseudo} \left( \omega \right))}}{{d\left( {V_{ac} } \right)}}}\right) - \frac{{dx_{{EL_{l} }} }}{{dV_{ac} }}|V_{dc = 0,} \omega_{b}$$
  When lateral PFM measurements are carried out on 2D layers on the dielectric substrates like SiO_2,_ dielectric screening, and charge injection effects have to be considered. It is hard to differentiate the actual piezoresponse from the effects of substrate charges accumulated on the dielectric and uncertain doping (explained in Sect. 10 of SI).
  Figure 5a compares measured piezoelectric coefficients before subtracting various background contributions and the effective piezoelectric coefficients calculated using Eq. (6) for monolayer MoS_2_ near the contact resonance frequency range. Here, the measured *d*_11_ is normalized with the gain corresponding to the operation frequency, and the electrostatic contributions are nullified to estimate the *d*_11*eff*_. The effects of the strain gradients in the out-of-the-plane direction are not significant for the measurements presented here. Strain gradient inducing the piezo effect is called the converse flexoelectric effect^37^. If the strain gradient in the z-direction (3) affects the measured in-plane piezoresponse, then the flexoelectric coefficient has to be non-zero when the electric field is applied in the x-direction (1). But, based on the crystal symmetry of MoS_2_, those elements are zero and consequently do not affect the in-plane piezoelectric response. These effects from the tip in the vertical direction are unlikely to affect the lateral PFM with in-plane field excitation.

**Figure 5:**
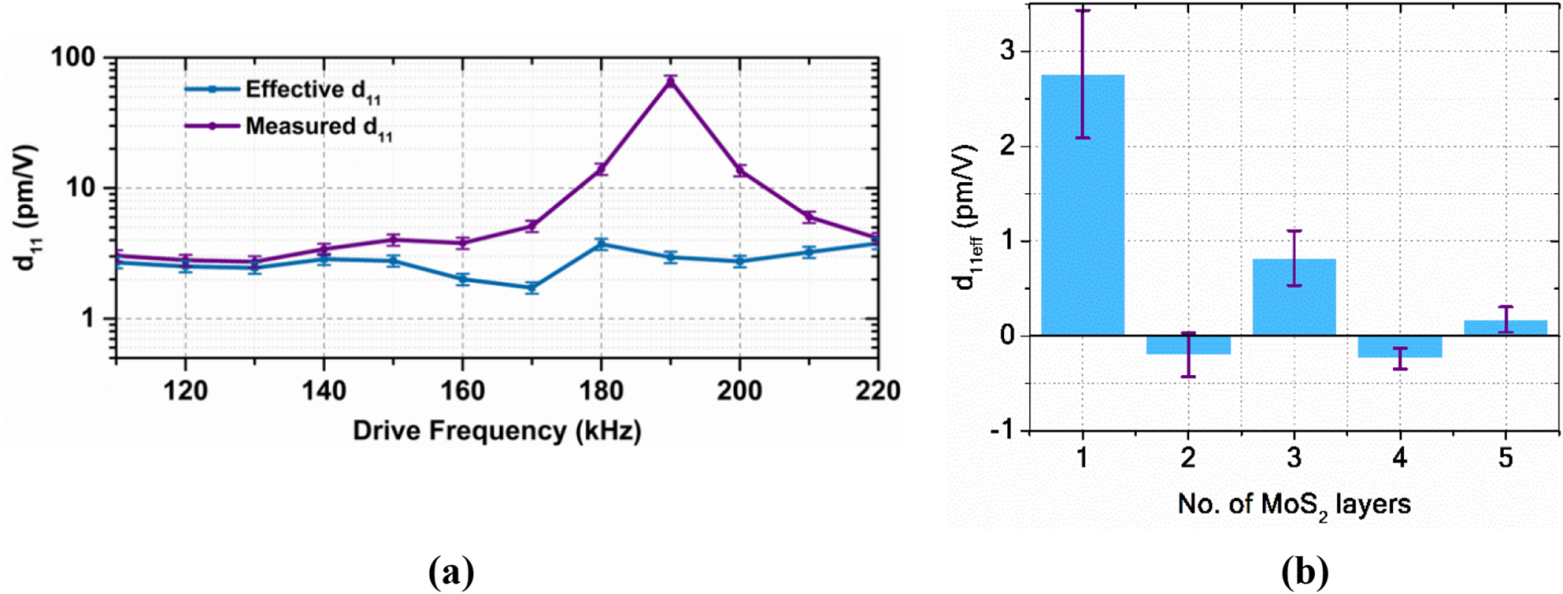
(**a**) The effective and measured *d*_11_ coefficients measured at different frequencies for a monolayer MoS_2_ near the contact resonance frequency range (here the gain in the signal by operating at a frequency within resonance bandwidth is normalized for effective piezoelectric coefficients), (**b**) d_11_ coefficients of MoS_2_ from monolayer to 5-layers.
  Figure 5b shows the estimated piezoelectric coefficients for 1–5 layers of MoS_2_. Refer to Sect. 11 of the SI for the measured and effective d_11_ plots for 2–5 layers of MoS_2_ near contact resonance. Samples with an odd number of layers of MoS_2_ have finite *d*_11_, whereas the ones with an even number of layers have an effective piezoelectric response indistinguishable from the background. This is because of the presence of an inversion centre in the samples with an even number of layers (D_6h_ symmetry). Piezoelectric coefficient (*d*_11_) of monolayer MoS_2_ obtained using this method are close to the theoretical coefficients estimated using density functional theory calculations by Duerloo et al.^1^ Further, to verify the lateral PFM methodology, the procedure is repeated on the AT-cut quartz crystal (see Sect. 12 SI).

## Conclusion

In conclusion, we have demonstrated the use of the lateral PFM technique with in-plane field excitation for the quantitative measurements of in-plane piezoelectric coupling coefficients. This lateral PFM method involves quantitative measurements at contact resonance, which is also useful for the other PFM measurements where the detection sensitivity is limited at off-resonance frequencies. The effective *d*_11_ piezoelectric coefficients measured using this technique for 2D-MoS_2_ layers ranging from monolayer to five layers of thickness matches well with the values predicted by theoretical models. The measurement technique has been verified by measuring the in-plane piezo-response on AT-cut quartz crystal. We also present methods to quantify the frequency-dependent background signals that arise in the measurement system. Further, MoS_2_ with an odd number of atomic layers have shown the piezoelectric effect, which agrees with the phenomenon that only non-centrosymmetric crystal structures exhibit piezoelectricity.

## Methods

### Materials

Molybdenite crystal is purchased from Graphene Supermarket. MoS_2_ layers are mechanically cleaved from this using scotch tape. Devices are fabricated on Si/SiO_2_ wafers with SiO_2_ thickness of 285 nm; a detailed process can be found in SI. SCM-PIC V2 AFM tips used for the measurements are purchased from Bruker.

### Measurements

Bruker dimensions ICON AFM instrument is used for PFM measurements; it has an internal built-in lock-in amplifier for the PFM mode to measure the amplitude of the AC oscillations. LabRam HR instrument from Horiba is used for Raman spectroscopy. Zeiss Ultra 55 SEM is used for scanning electron microscopy images. Leica DM2500 optical microscope is used to take optical micrographs.

## Supplementary Information

Supplementary Information
