# Supplementary material for: Quantitative probe for in-plane piezoelectric coupling in 2D materials
Source: Sci Rep. 2021 Mar 29;11:7066. doi: 10.1038/s41598-021-86252-9 (PMC8007818; doi:10.1038/s41598-021-86252-9)
Supplement: Supplementary file 1 — Supplementary Information [file 41598_2021_86252_MOESM1_ESM.pdf]

SUPPORTING INFORMATION

Quantitative probe for in-plane piezoelectric coupling in 2D materials

Sai Saraswathi Yarajena[1*], Rabindra Biswas[2], Varun Raghunathan[2], Akshay K. Naik[1]*

Centre for Nano Science and Engineering[1], Department of Electrical Communication Engineering[2], Indian Institute of Science, Bengaluru, India, 560012

*corresponding author: anaik@iisc.ac.in

1. Device Fabrication

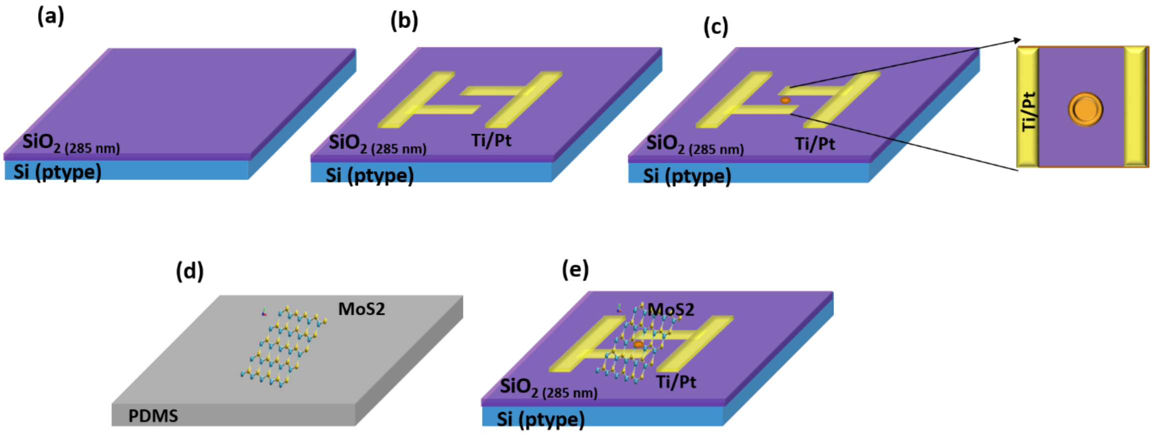

Figure S1: Simplified Process flow for the fabrication of devices. Detailed description for each of the process step depicted here is explained as follows in steps from a) to e) respectively.

a) Devices are fabricated on a p-type silicon substrate (Resistivity 0.005 ohm-cm) with 285 nm thickness of Silicon dioxide (SiO_2).

b) Initially, metal contacts are patterned on the substrate. To achieve this, S1805 optical resist is spin-coated on the substrate, and the photolithography process is carried out with the direct writing technique (μpg 501 Heidelberg tool). Then, metallization of Platinum (Pt) contacts with Titanium (Ti) as adhesion metal layers with the thickness of 15/50 nm is carried out using Techport e-beam evaporator. This is followed by a lift-off process in acetone.

c) To make a circular trench in between the electrodes, PMMA resists (495 A4 and 950 A2) are spin-coated and a circular opening is formed between the contacts using the e-beam lithography technique (Raith eline tool). SiO_2 is completely etched away in the circular drum portion by using Reactive ion etching (RIE from Oxford instruments) followed by buffered hydrofluoric acid etch.
d) 2H-MoS_2 flakes are cleaved from the molybdenite crystal on to scotch tape. Then they are mechanically exfoliated on the PDMS (Polydimethylsiloxane) film. Thin flakes are identified by the optical contrast of the flakes. The number of layers in these 2d-layers is confirmed using Raman spectroscopy (Horiba LabRam HR).
e) These selected flakes are transferred on to the pre-patterned substrate using dry transfer technique[1].

2. Characterization using optical microscope and AFM

Fig. S2(a) shows the AFM topography (height) image of the MoS_2 suspended monolayer device. This image is taken in contact mode of AFM. If the MoS_2 is suspended on the circular trench, then the line profile of the height image across the trench region resembles as shown in fig. S2(b). Measured depth near the circular drum region should be much less than the value of actual trench depth (285 nm) when MoS_2 is suspended in that region. The depth measured across the trench region is the addition of indentation depth because of the applied normal force while scanning and the initial sag of the MoS_2 layer. Fig. S2(c) shows the image of the same monolayer device after it has collapsed (after multiple measurements and SHG). When the MoS_2 layer is collapsed on the trench, the line profile across the trench is shown in fig. S2(d) and the depth measured at the circular trench region is close to 285nm, which is the actual etch depth.

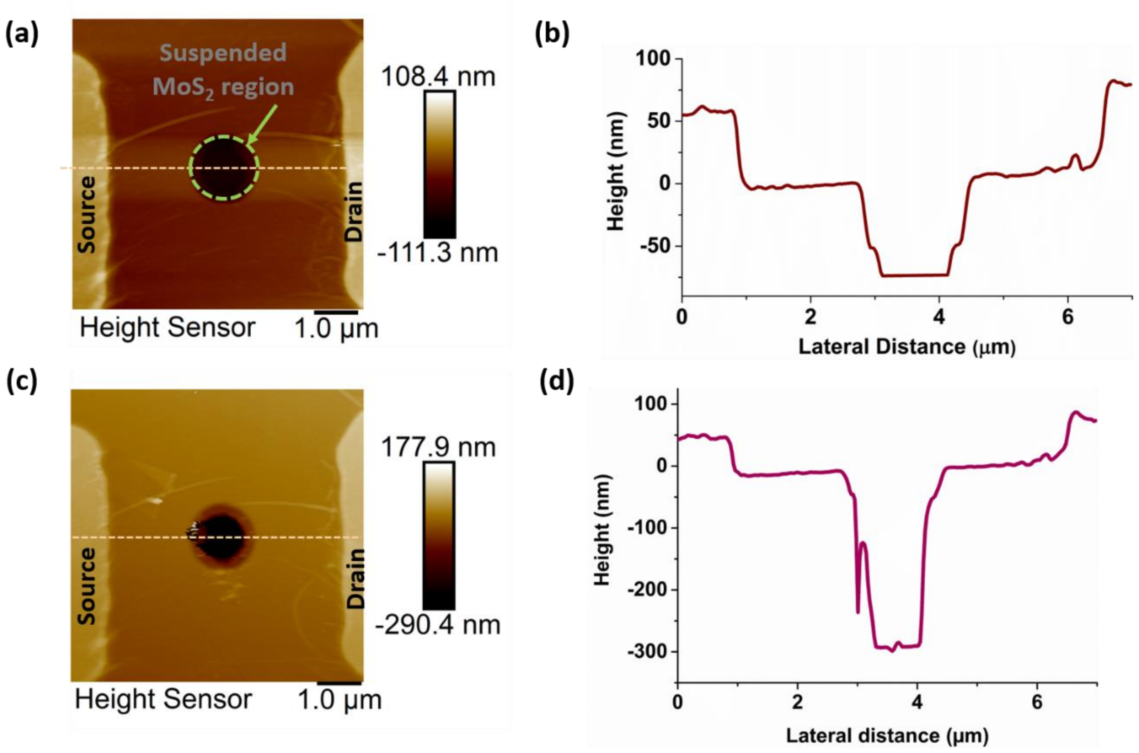

Figure S2: (a, b) AFM topography of MoS_2 suspended monolayer device and the corresponding line along the line indicated in (a). (c, d) AFM topography of the device when it has collapsed in the trench and the corresponding line profile along the line indicated in (c).

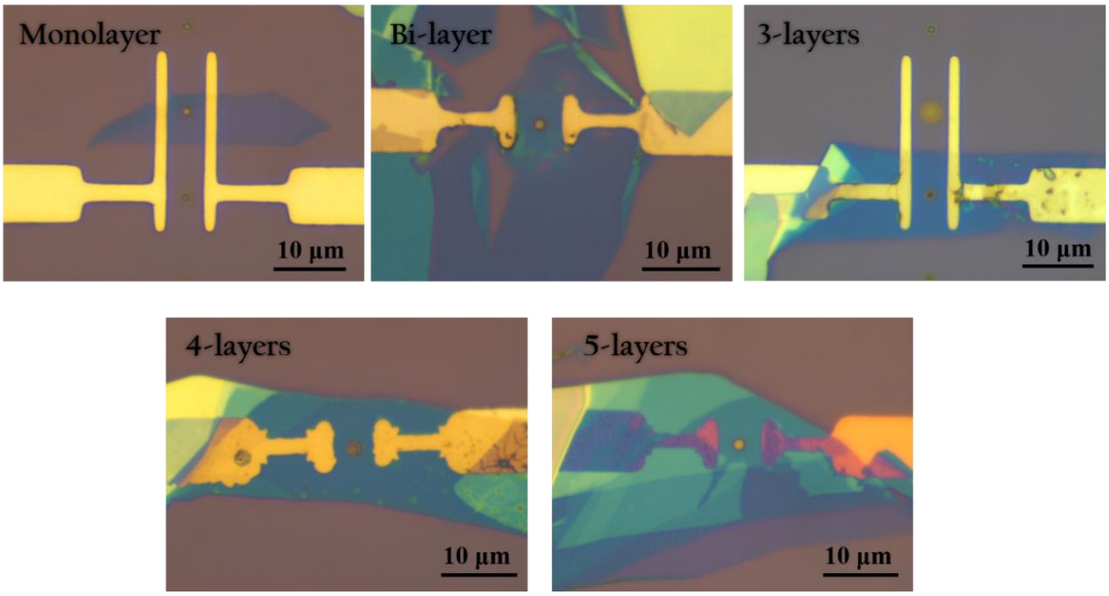

Figure S3: Optical micrographs of 2D-MoS_2 devices with number of layers varying from monolayer to 5-layers

3. Second harmonic generation microscopy (SHG)

For d_{11} measurement, the direction of the electric field must be along the armchair edge of the MoS_2 flake. The direction of the metal pads with respect to the flakes is found based on the polarised SHG studies. SHG experiments are carried out to find the edge chirality of the flakes. A linearly polarised femtosecond pulsed laser operating at 1040 nm is used for the measurements, and the corresponding second harmonic component is detected at 520 nm. An optical image collected at 1040 nm (incident wavelength) is used to locate the region of interest from the sample (fig S4(a)). Fig. S4(b) shows the SHG mapping of the monolayer MoS_2 flake. It is observed that SHG intensity is relatively high on the circular drum region when MoS_2 is suspended in that region. It is also observed that if MoS2 is collapsed in the circular drum region, SHG intensity vanishes in some samples. Fig. S4(c) shows the plot with SHG intensity vs. incident power. SHG power shows quadratic dependence with the input power.

The second harmonic field amplitude of the parallel component is given by[2]

$$E_{2\omega} = C\chi^{(2)}_{xxx}\cos(3\phi + \phi_0) \tag{S1}$$

Where ω is the incident laser frequency, 2ω is the second harmonic frequency, C is the proportionality constant which is a function of electric field component at ω (E_ω) and the dielectric environment. The direction 'x' corresponds to the armchair direction of the 2D-MoS_2 flake, ϕ is the sample rotation angle w.r.t the laboratory x-coordinate of the experimental system and ϕ_0 is the initial crystallographic orientation of MoS_2 flake. Fig S4(d) shows the polar plot of the polarised SHG data collected at different rotation angles, here laser is polarized along the y-axis. By fitting the intensity(I) plot to $I \alpha E^{2,}$ we get ϕ_0 as 0^0 (fitting error of the polar plot has a standard deviation of 2^0). Hence the armchair direction of the monolayer MoS_2 flake is identified along the electrodes. Electrodes for two suspended monolayer MoS_2 devices are along the armchair direction.

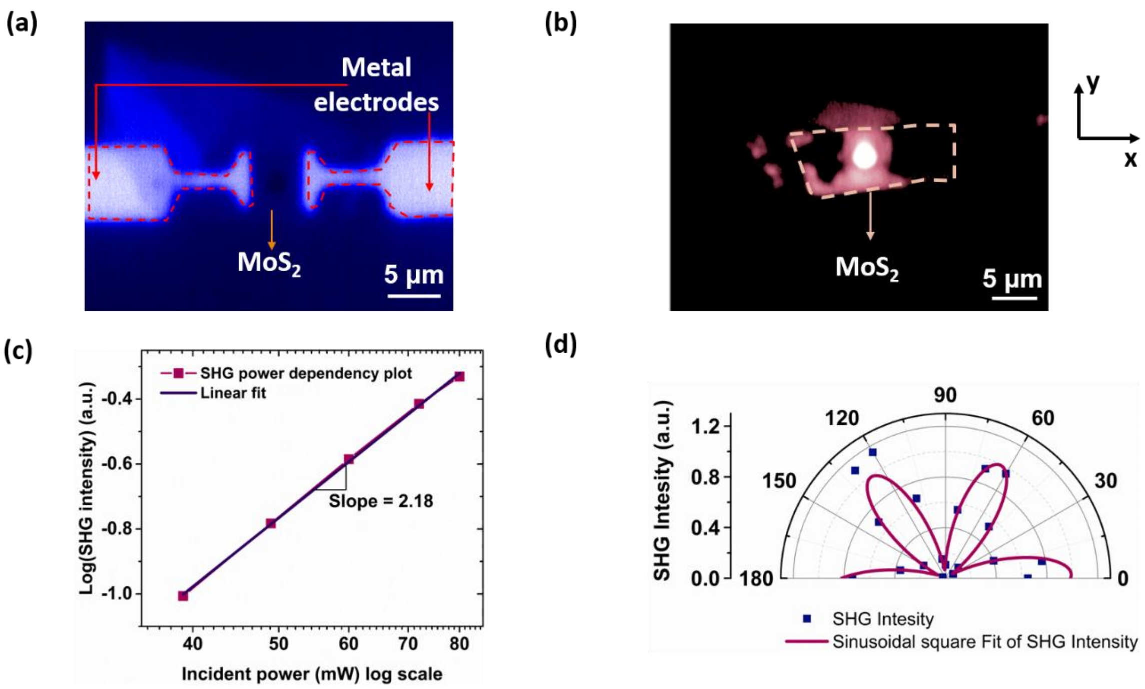

Figure S4: (a) Reflection microscopy image collected at incident laser wavelength i.e. 1040 nm (b) SHG amplitude mapping (at 520 nm) of one of the monolayer MoS_2 devices used for the piezo measurements. (c) SHG intensity as function of incident power. (d) Polar plot of the parallel component of the polarised SHG intensity plotted at various sample rotation angles w.r.t laboratory coordinate axes.

4. Vertical Deflection sensitivity calibration

To calculate the vertical deflection sensitivity of the AFM tip, force-distance curves are obtained on the sapphire substrate. Fig. S5(a) shows the deflection error plot when the tip is approaching and is retracting from the sample. The slope of the linear region in the approach/retract curve is defined as inverse vertical deflection sensitivity of the tip. From this plot, we calculate the vertical deflection sensitivity (n_d) of one of the SCM-PIC tips to be 207.5 nm/V. Vertical deflection sensitivity of the SCM-PIC tip is calculated using the force-distance method, and it is found to be 200+/-10 nm/V (n_d) for various tips used for the measurement. Fig S5(b) shows the thermal tuning data of the AFM cantilever (cantilever is not in contact with the sample, and it is free to vibrate in the air). The normal spring constant (k_N) of the cantilever is estimated from thermal tuning data[3]. The normal spring constant calculated from this is 0.17 N/m for one of the tips.

This information is needed to estimate the amount of normal force (F_N=k_N*n_d**setpoint voltage*) applied on the sample corresponding to the deflection voltage set point of the system. This force is maintained constant across all the piezo and pseudo measurements for a given sample.

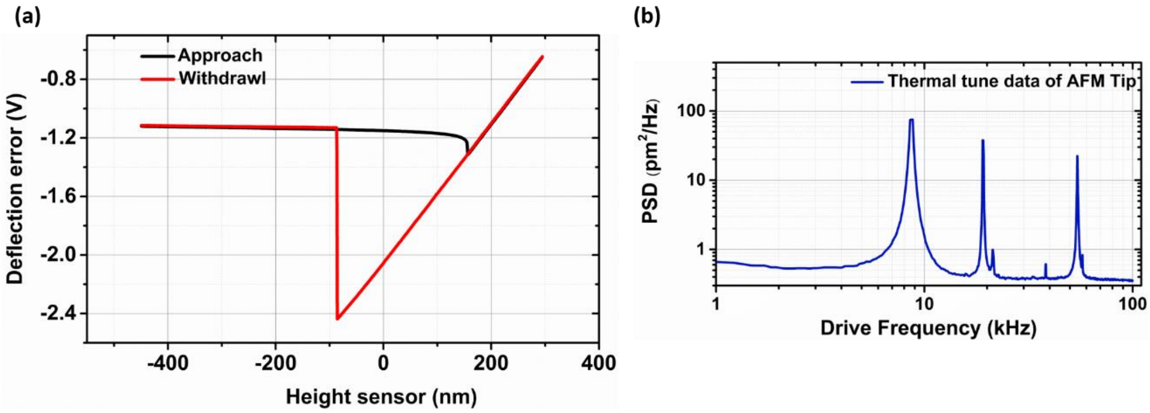

Figure S5: (a) Deflection vs. distance curve of SCM-PIC tip on the sapphire substrate, (b) Thermal tuning data for spring constant calibration

5. Torsional deflection sensitivity calibration

Angle conversion factor method proposed by Choi D et al. [4] is used to estimate the lateral deflection sensitivity. For this calibration procedure, a standard silicon grating sample from Bruker with a step height of 180nm is used. Moment balance equation when the tip is on the flat portion of the surface and while climbing up at the apex is given by equations S2 and S3, respectively. The angles θ_0 and θ_1 are the twist angles of the AFM cantilever (see fig. S6(a, b)) when it is on the flat surface and when climbing up the edge, respectively.

$$-\mu H N_s - H\theta_0 N_s - k_t\theta_0 = 0 \qquad \text{(S2)}$$

$$-HN_s[\theta_1 + (\frac{cos\alpha + \mu sin\alpha}{sin\alpha - \mu cos\alpha})] = k_t\theta_1 \qquad \text{(S3)}$$

where, k_t -Torsional spring constant, μ- coefficient of friction, $H=h+t/2$, h- is the height of the tip, α- half the tip side angle and N_s-Normal force

The angle conversion factor η relates the lateral deflection voltages and the twist angles of the cantilever.

$$\theta_0 = V_0.\eta \qquad \theta_1 = V_1.\eta \qquad \text{(S4)}$$

The torsional spring constant is given by

$$k_t = \frac{GWt^3}{3L} \quad \text{(S5)}$$

Where G is the shear modulus of the cantilever, W, L, t are the width, length, and thickness of the cantilever, respectively.

Solving the equations S2 to S5, we get equation S6, which relates the applied normal forces with twist angles

$$\theta_0^2 - \frac{\theta_0 tan\alpha . N_s(a-1)}{a(b+N_s)} - \frac{{N_s}^2}{a(b+N_s)^2} = 0 \quad \text{(S6)}$$

where $$a = \frac{V_1}{V_0} \qquad b = \frac{k_t}{H} \quad \text{(S7)}$$

To determine the lateral deflection sensitivity for the AFM probes, the tip is scanned laterally across the standard silicon step gratings (step height s=180 nm) as shown in the schematic below (fig. S6(a,c)). Friction images are captured in LFM mode (Lateral force microscopy) by keeping applied normal force constant (fig. S6(d)). When the tip climbs up along the step grating, it experiences a maximum twist angle at the apex and fig. S6(b) shows the lateral deflection voltage signals. The peak voltage read while the tip is climbing is termed V_1, while V_0 is the average deflection signal when the tip is moving on the flat surface. This procedure is repeated at various normal forces ranging from 20nN to 60nN of force. Fig. S6(e) shows the plots of V_0 respectively at variable normal forces, and S6(f) shows the plot of calculated θ_0 from equation S6 at a given normal force. Angle conversion factor $'\eta'$ is the ratio of the slopes of these two curves.

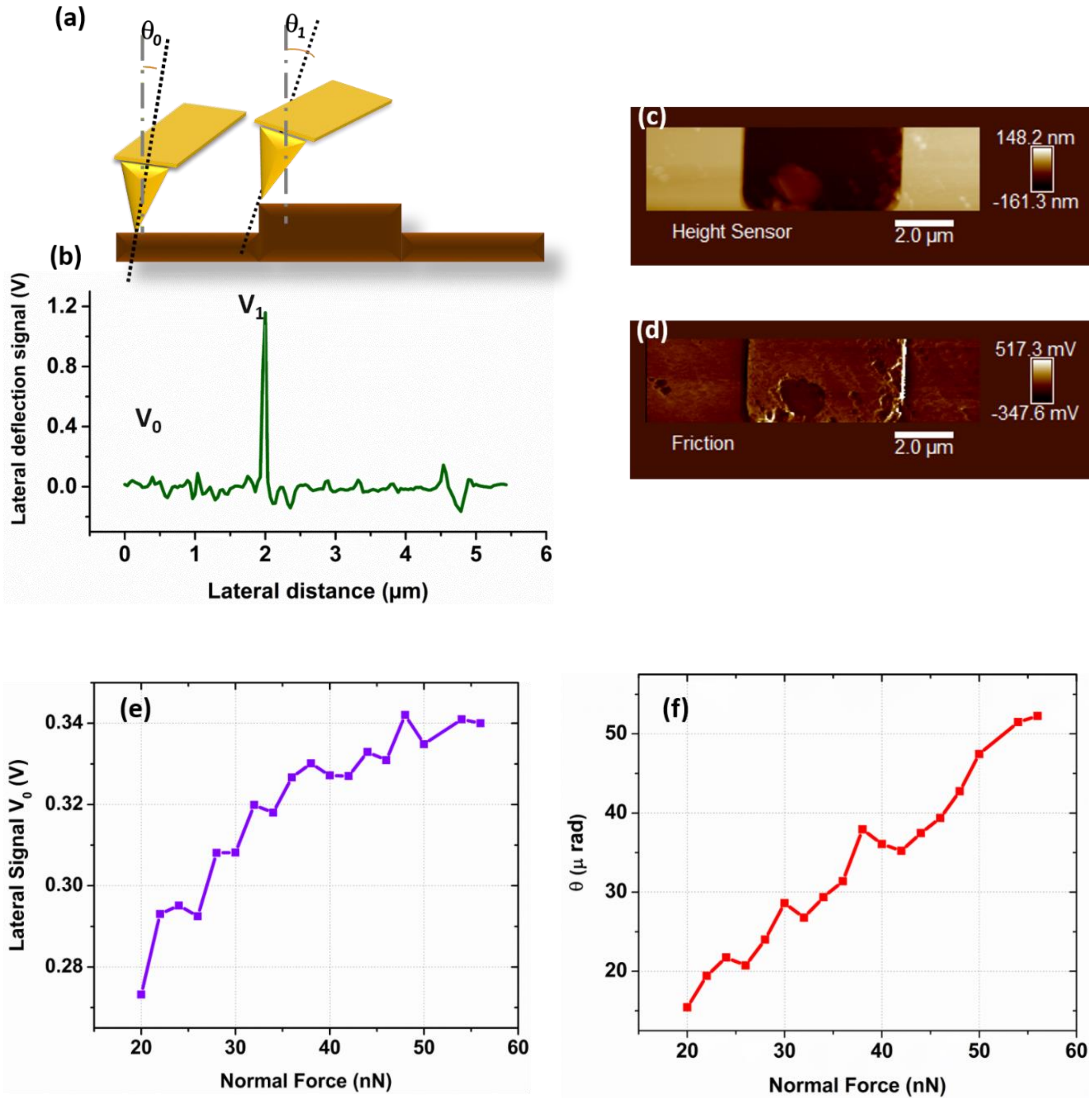

Figure S6: (a) schematic depicting cantilever deflection angles when the tip is climbing up. (b) lateral deflection voltage seen by PSPD when the tip is scanning laterally, (c) AFM topography image of the silicon grating used for calibration. (d) Corresponding friction image when the tip is scanning the area. (e)Plot showing lateral deflection varying with the applied normal force. (f) Estimated twist angle variation on the flat surface with the applied normal force.

Lateral spring constant relevant to these measurements when the AFM tip end is in contact with the sample is given as [5,6]

$$k_l = \frac{k_t}{((h + \frac{t}{2})^2} \tag{S8}$$

This lateral spring constant is not to be confused with the true lateral spring constant of the tip, which is related to the stiffness of the cantilever and corresponding true lateral deflections[6]. When the tip edge is

interacting with the sample in contact mode of AFM, the PSPD relates the lateral deflections of the cantilever related to k_l given by eq. S8.

Lateral deflection sensitivity factor l_d can be calculated from the angle conversion factor as follows

$$l_d = \eta * (h + (t/2) - s) \qquad \text{(S9)}$$

Where h is the height of the AFM tip, t is the thickness of the cantilever, and s is the height of the silicon grating step. These are measured from the scanning electron micrographs of the tip taken by placing it on a stub (holder) used for cross-section imaging (fig. S7(a) & S7(b)). SEM images are taken before and after the measurements to check if the tip is intact post measurements. Shear modulus(G) for the calculations is taken as 60Gpa for silicon cantilevers. Geometrical parameters measured from the SEM images and estimated parameters for some of the tips used for the measurements are tabulated in Table S1.

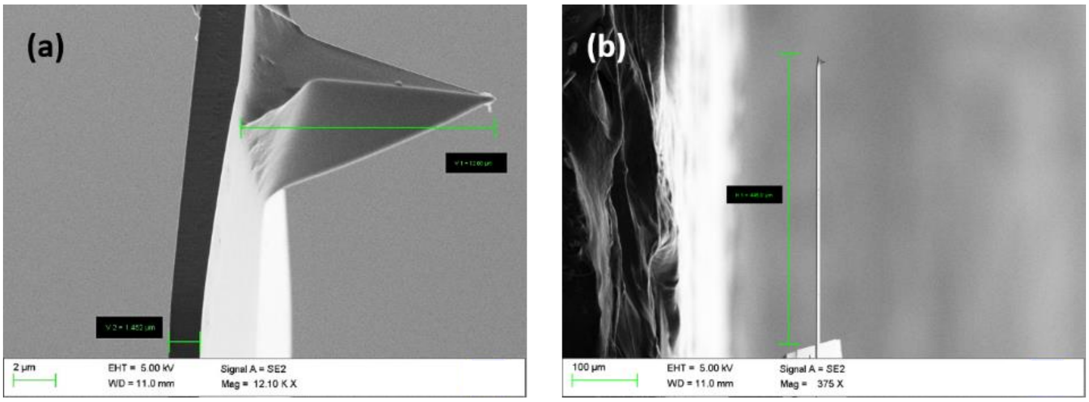

Figure S7: (a) SEM images of the tip near the tip end. (b) SEM micrograph to calculate the length of the cantilever.

**Parameters**	**PIC_5**	**PIC_7**	**PIC_10**	**PIC_n1**	**PIC_Bruker**
**Cantilever Length (*L*) in µm**	450	445	442.6	440	450
**Cantilever Width (*W*) in µm**	37	38	37	35	35

**Cantilever Thickness (*t*) in μm**	1.5	1.41	1.43	1.478	1.8
**Tip Height (*h*) in μm**	11.9	12	12.43	10.89	15
**Torsional Spring constant (K_t) in nNm/rad**	5.5	4.79	4.89	5.13	9.07
**Lateral Spring constant (K_l) in N/m**	34.6	39.72	28.35	37.98	35.8
**Angle conversion factor (η) in μrad/V**	779.95	906.18	918.1	774.71	599.76
**Lateral deflection sensitivity (l_d) in nm/V**	12.26	14.24	14.43	12.17	9.428

Table S1: Geometrical parameters of various AFM probes extracted from the SEM images and the derived parameters for the LPFM measurements.

6. Comparison of obtained lateral deflection in the current configuration with alternative configuration A

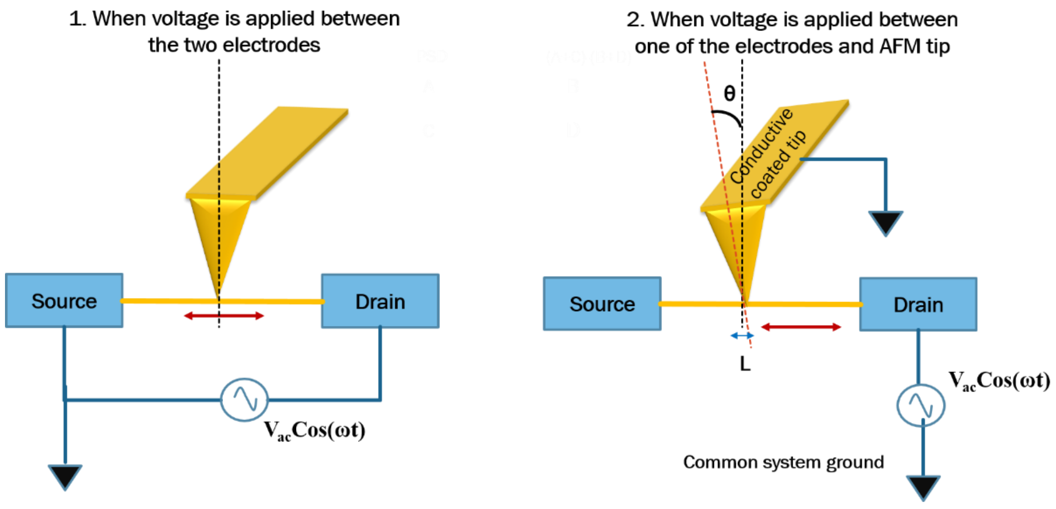

(a) (b)

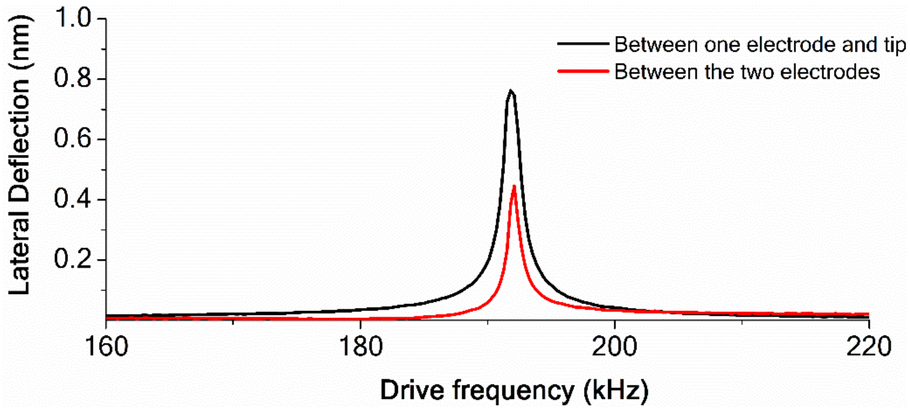

(c)

Figure S8: Lateral deflection measurements when the voltage is applied between the two electrodes and when the voltage is applied between one electrode and the tip with V_{ac}=3V

When the voltage is applied between the source and the drain electrodes (fig. S8(a)), because of the in-plane piezoelectric effect in MoS_2, the net lateral displacement of the AFM tip is zero when the tip is placed at the center. Even if the tip is not placed precisely at the center, we do not get optimal results. Whereas in the current scheme (fig. S8(b)), the tip can detect net lateral deflection in one direction so that the effective displacement can be measured. It can be observed from fig. S8(c) that the signal strength is much higher when voltage is applied between the electrode and the tip in contact with the sample when compared to the signal obtained when voltage is applied between the two electrodes. The observed signal in that case (fig. S8(b)) was much smaller, and it is comparable to the pseudo piezoresponse (Fig 4(c) of the manuscript).

7. Instrumental noise floor in the measurement frequency range

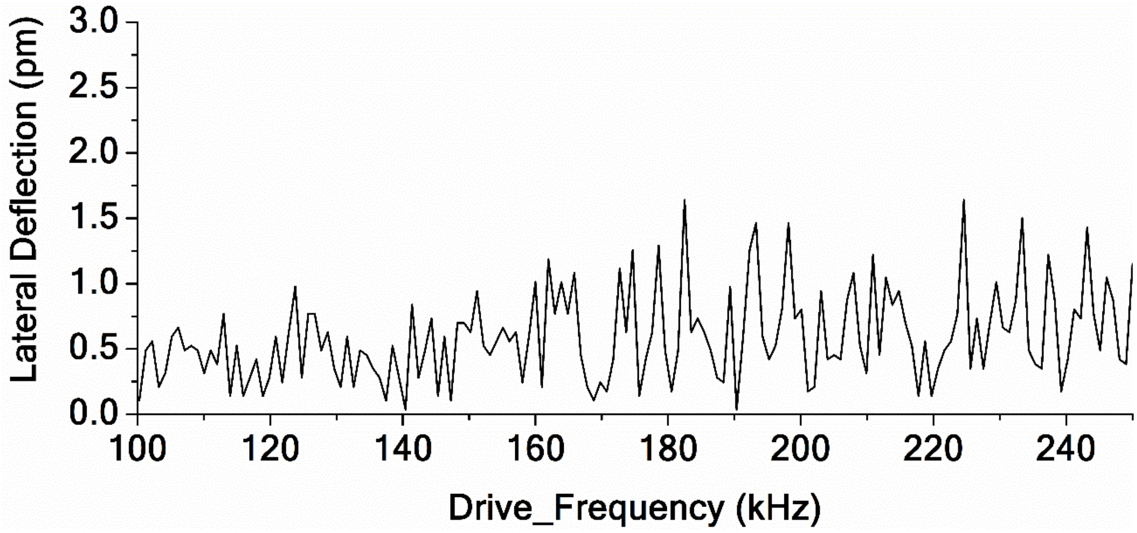

Figure S9: Equivalent lateral deflection for the particular tip in the instrument setup when the tip is not in contact with the sample (K_BT/q~ 26mV)

From the Fig. S9 average lateral deflection in the given frequency of operation is measured as 0.59 pm. This is considered as instrument noise floor for the measurement of lateral deflection. Contact resonance can enhance the signal levels from pico meter to nano meter scale (refer Fig. S9) and thus enables the measurement of low piezoelectric coefficients.

8. Selection of the torsional resonance frequency range for monolayer MoS_2 Device

In the manuscript, a specific drive frequency range (110 kHz to 220 kHz) is selected to discuss operation of the lateral PFM near the torsional resonance. To identify the peak frequency, we fit a Lorentzian equation to the drive frequency response of the lateral piezoresponse curve. Then, the identified local maxima position of the data is taken as peak frequency datapoint. A constant baseline is chosen from the local minima which is based on the statistical median of the amplitude data near the torsional resonance peak. The x-coordinates (frequencies) corresponding to the intersection of the baseline with the experimental data are chosen as base frequencies. Identified peak and base frequency positions are indicated in Fig. S10 (a). Fig S10(b) shows the Lorentzian peak fit of the torsional resonance peak. For this particular device, the resonance peak is near 190 kHz (vertical line marked in red), and base frequency positions are near 100 and 220 kHz frequencies (vertical lines marked in blue). From this analysis, the base frequency (ω_0) is selected at 100 kHz for the measurements. Hence, the operating frequency range is selected above the baseline frequencies, i.e. from 110 kHz to 220 kHz, to study lateral PFM near torsional resonance.

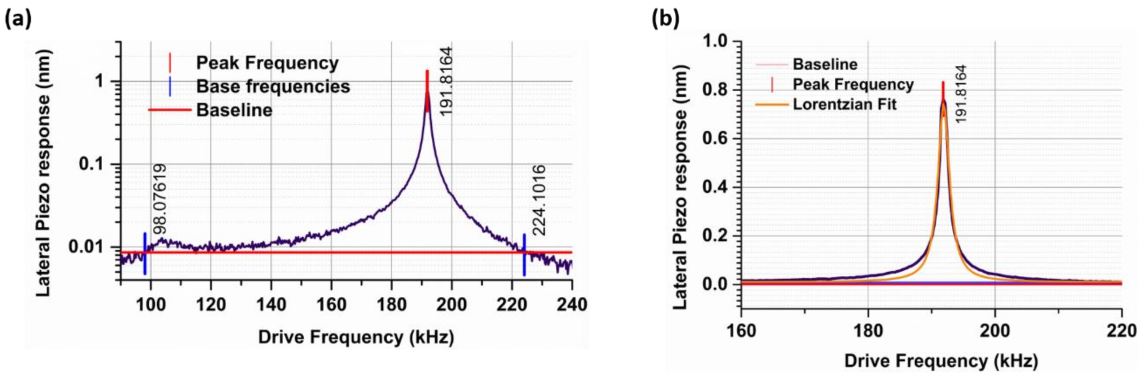

Figure S10: (a) Drive frequency sweep curve when AFM tip is in contact with a suspended monolayer of MoS_2 showing the baseline and peak markers. (b) The Lorentzian fit of the drive frequency curve for the torsional resonance peak. This data is taken with applied drive amplitude (V_{ac})=3V.

9. Contribution from the Pseudo piezo response

In the current measurement scheme, d_{11}_pseudo contributes to the pseudo piezo response to the measured d_{11} coefficient. Fig. S11 shows the $d_{11pseudo}$ measured near the torsional resonance frequency range. Here, the pseudo response to the d_{11} is compared with the measured and effective d_{11} coefficients. $d_{11pseudo}$ measured near contact torsional resonance peak (190 kHz) is ~0.8pm/V for this set of measurements. From this, we emphasize that pseudo response contribution becomes significant for estimating the coefficients in the sub pm/V range.

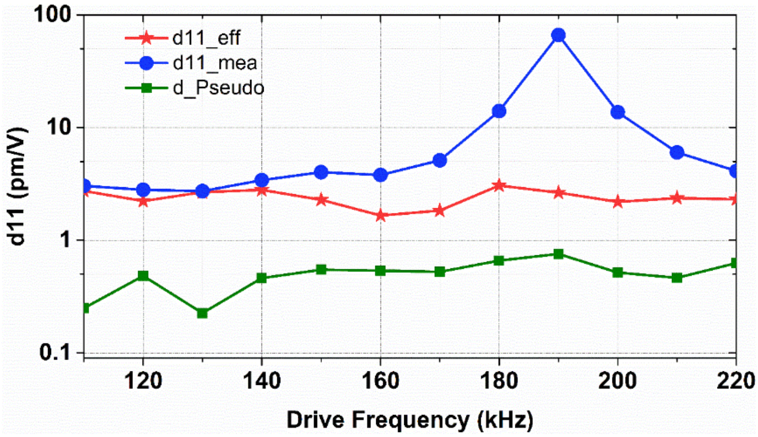

Figure S11: Plot showing various contributions to Lateral Piezoresponse for Monolayer MoS_2

10. Comparison of Piezoresponse for suspended and on substrate MoS_2 monolayers

We have fabricated a device wherein a monolayer MoS_2 (confirmed from Raman data) is transferred onto the SiO_2/Si substrate without a trench. Fig. S12(a), (b) shows the lateral deflection amplitude mapping image and the topography image, respectively, for the monolayer on the substrate (a dashed line indicates monolayer region). Similar measurements performed on monolayer MoS_2 on a trench are shown in Fig. S12(c) and (d). It is clear that when the MoS_2 is on the substrate, we are not able to distinguish the deflection signal from the monolayer MoS_2 and signal from the substrate. On the other

hand, the lateral deflection mapping image from Fig. S12(c) indicates that the lateral deflection amplitude is higher in the suspended region.

Fig S12(e) shows the comparison of the lateral deflection amplitude as a function of drive amplitude for MoS_2 on the suspended region of figure S12(d) (green line), for MoS_2 on the SiO_2 substrate at point A in Fig. S12(b) (black line) and directly on the SiO_2 at point B in Fig. S12(b) (red line). The plots are obtained after normalizing for resonance frequency gains. Again, it is clear that the lateral deflection signal from the monolayer MoS_2 is harder to distinguish from the substrate unless the measurements are performed on suspended portions of the MOS_2.

Note: The amplitude mapping images presented in Fig. S12(a & c) are for the qualitative illustration. The amplitude mapping images are acquired using the line scan, and relatively less contact force is applied to avoid damage to the tip. Furthermore, to acquire the amplitude mapping images shown in fig. 12(a & c), we use relatively less contact force. The smaller force ensures that the tip does not get damaged while scanning and avoids a short circuit when the tip is in contact with the drain electrode.

In Fig. S1(e), the data is obtained from a single point at constant applied force. Quantitative information is extracted from the point measurements.

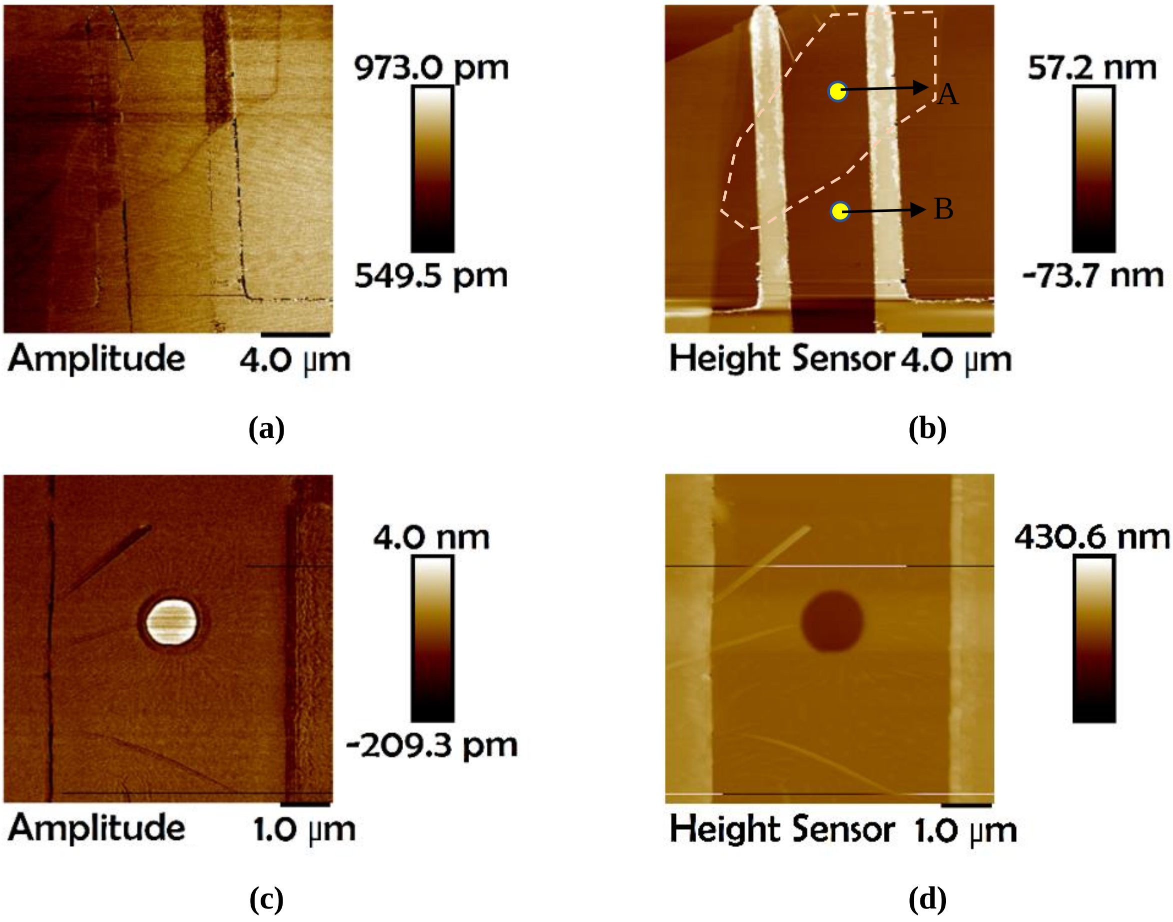

(a) **(b)**

(c) **(d)**

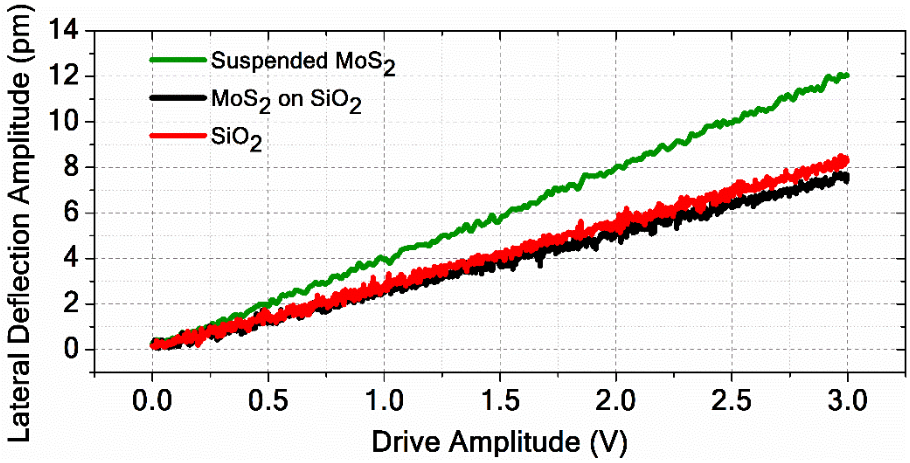

(e)

Figure S12: (a) Lateral deflection amplitude/piezoresponse and (b) corresponding topography image of a monolayer device on SiO_2/Si substrate. Operating conditions: V_{ac}=3V at 180 kHz (below contact resonance for this sample-tip system). (c) Lateral deflection amplitude/piezoresponse and (d) corresponding topography image for monolayer MoS_2 on a trench. Operating conditions: V_{ac}=3Vat 170 kHz (below contact resonance for this sample-tip system). (Note: Different gains involved in the system are not normalized). (e) Comparison of the Piezoresponse on suspended MoS_2 monolayer (green line), MoS_2 monolayer on the substrate (black line), and on the Si/SiO_2 substrate (red line). The displacements have been normalized by the gains of each measurement.

11. d_{11} coefficient for different layers of MoS_2

Fig. S13 shows the measured and effective d_{11} coefficients for MoS_2 of 2 to 5 layers calculated using the methods discussed in the manuscript. It can be observed that the frequency bandwidth of the resonance is wider for the mono and 3-Layers when compared to even number of layers. This indicates that lateral piezoelectric response in odd no. of layers of MoS_2 enhances the torsional resonance.

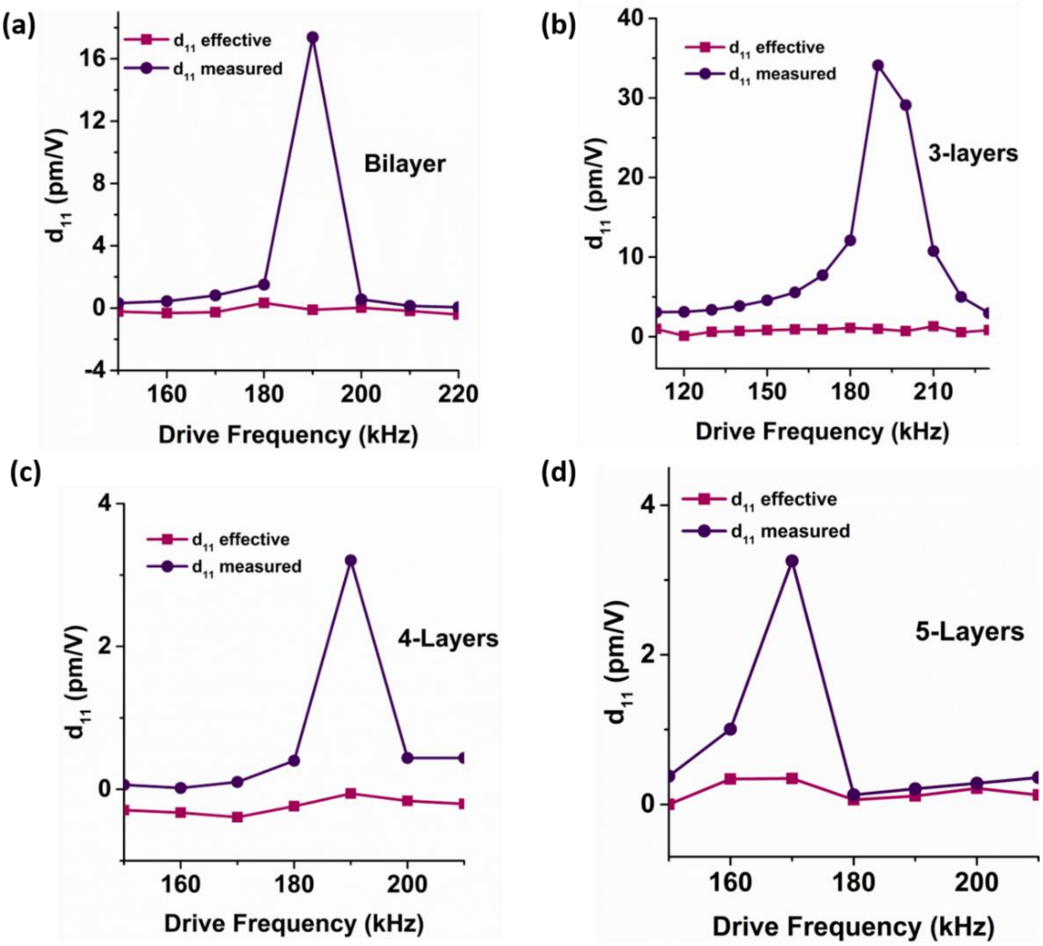

Figure S13: Piezo responses obtained on (a) Bilayer (b) 3-layers (c) 4-layers and (d) 5-layers

12. LPFM on quartz

To test if these methods can be applied to the bulk samples, LPFM is carried out on a quartz substrate to find the lateral piezo coefficient. Fig. S14(a) shows the XRD plot of the AT-cut quartz crystal, which confirms the crystalline behaviour of the quartz. Metal electrodes (Cr/Au 10/100 nm) are patterned on the surface quartz substrate as shown in Fig. S14(b). The in-plane piezo coefficient for AT-cut quartz crystal was found to be 1.6 -2.1 pm/V, which is close to other experimentally verified values, i.e., 1.9 pm/V[7] (Note: Pseudo-piezoresponse is not separated here as the metal electrode is also on quartz). Fig. S14(c) shows the lateral piezoresponse obtained on the quartz sample at its base frequency of resonance. Fig. S14(d) shows the piezoresponse obtained on quartz sample at various drive frequencies(ω) at constant drive amplitude (V_{ac}).

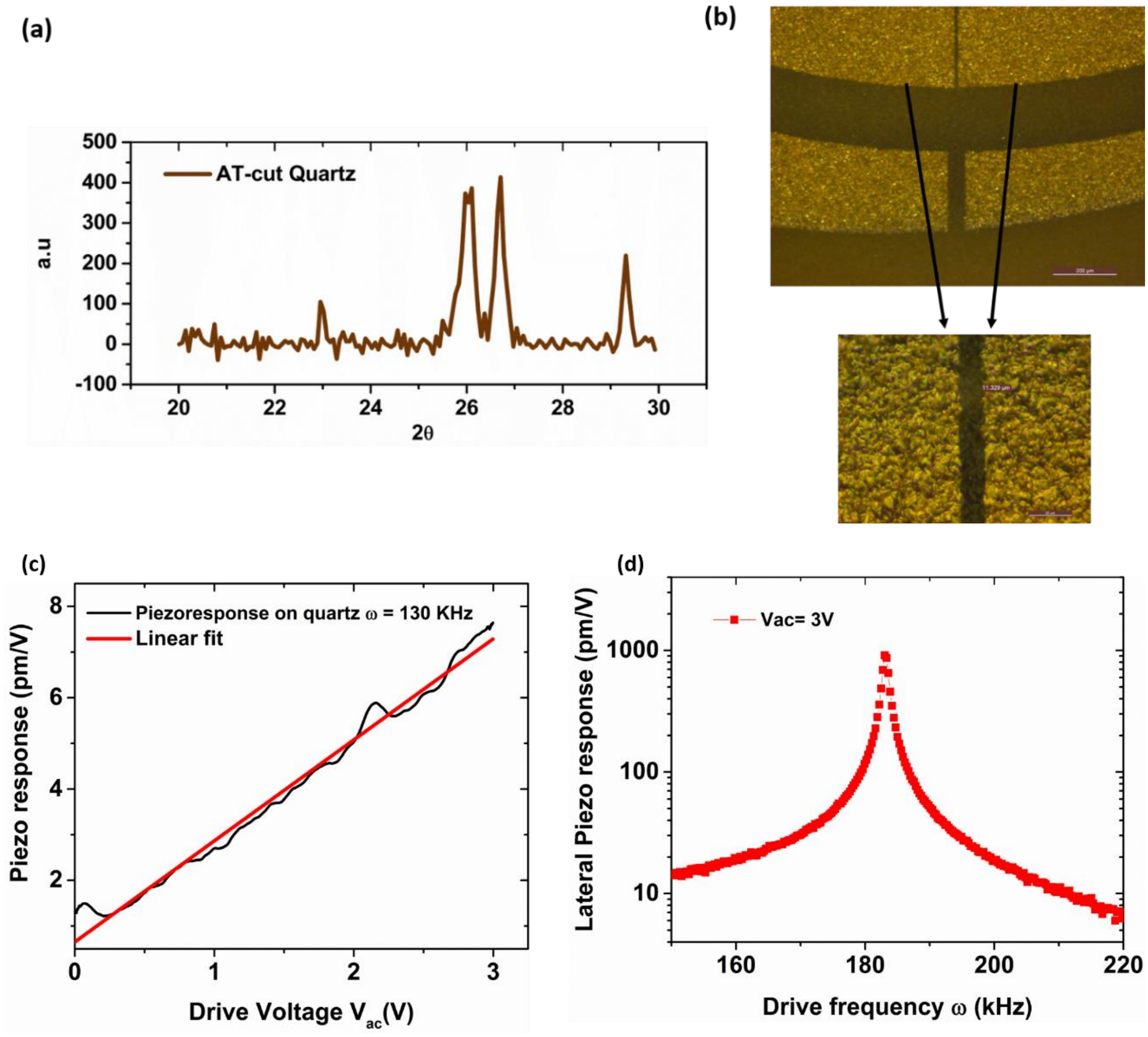

Figure S14: (a) XRD plot of AT cut quartz crystal, (b) Optical micrograph of quartz crystal with electrodes deposited on the top, (c) Piezoelectric response measured near its base frequency (d) Lateral piezoelectric response observed at variable drive frequencies at constant ac drive.

References:

1. Castellanos-Gomez, A. *et al.* Deterministic transfer of two-dimensional materials by all-dry viscoelastic stamping. *2D Mater.* **1**, 11002 (2014).
2. Malard, L. M., Alencar, T. V, Barboza, A. P. M., Mak, K. F. & De Paula, A. M. Observation of intense second harmonic generation from MoS 2 atomic crystals. *Phys. Rev. B* **87**, 201401 (2013).
3. Hutter, J. L. & Bechhoefer, J. Calibration of atomic-force microscope tips. *Rev. Sci. Instrum.* **64**, 1868–1873 (1993).
4. Choi, D., Hwang, W. & Yoon, E. Improved lateral force calibration based on the angle conversion factor in atomic force microscopy. *J. Microsc.* **228**, 190–199 (2007).
5. Munz, M. Force calibration in lateral force microscopy: a review of the experimental methods. *J.*

Phys. D. Appl. Phys. **43**, 63001 (2010).

6. Parkin, J. D. & Hähner, G. Calibration of the torsional and lateral spring constants of cantilever sensors. *Nanotechnology* **25**, 225701 (2014).
7. Tiersten, H. F. Thickness Vibrations of Piezoelectric Plates. *J. Acoust. Soc. Am.* **34**, 718 (1962).
